# Distinct temperature sensitivity of soil carbon decomposition in forest organic layer and mineral soil

**DOI:** 10.1038/srep06512

**Published:** 2014-10-01

**Authors:** Wenhua Xu, Wei Li, Ping Jiang, Hui Wang, Edith Bai

**Affiliations:** 1 State Key Laboratory of Forest and Soil Ecology, Institute of Applied Ecology, Chinese Academy of Sciences, Shenyang 110164, China; 2 University of Chinese Academy of Sciences, Beijing 100049, China

## Abstract

The roles of substrate availability and quality in determining temperature sensitivity (*Q*_10_) of soil carbon (C) decomposition are still unclear, which limits our ability to predict how soil C storage and cycling would respond to climate change. Here we determined *Q*_10_ in surface organic layer and subsurface mineral soil along an elevation gradient in a temperate forest ecosystem. *Q*_10_ was calculated by comparing the times required to respire a given amount of soil C at 15 and 25°C in a 350-day incubation. Results indicated that *Q*_10_ of the organic layer was 0.22–0.71 (absolute difference) higher than *Q*_10_ of the mineral soil. *Q*_10_ in both the organic layer (2.5–3.4) and the mineral soil (2.1–2.8) increased with decreasing substrate quality during the incubation. This enhancement of *Q*_10_ over incubation time in both layers suggested that *Q*_10_ of more labile C was lower than that of more recalcitrant C, consistent with the Arrhenius kinetics. No clear trend of *Q*_10_ was found along the elevation gradient. Because the soil organic C pool of the organic layer in temperate forests is large, its higher temperature sensitivity highlights its importance in C cycling under global warming.

Soil is the largest reservoir of terrestrial carbon (C) compared to vegetation and atmosphere^1^ and has diverse soil organic C (SOC) compounds with different stability^2^. Concerns about the climate–C cycle feedback under current global climate change have catalyzed efforts on SOC decomposition and its temperature sensitivity (*Q*_10_)^2^,^3^,^4^,^5^,^6^, which represents change in C decomposition rate to a 10°C temperature increase. However, as yet there is no consensus on the relationship of SOC decomposition with temperature owing to various stability of different SOC components^2^,^5^,^7^. As a result, doubts have been cast on the assumed similar sensitivities of discrete C pools to temperature in soil C models^8^.

Most uncertainty in predicting responses of C storage to changing climate comes from temperature sensitivity of recalcitrant soil C. Compared to more labile C, recalcitrant C has been found to have similar^4^,^9^ or lower^3^,^10^ *Q*_10_ of C decomposition. However, substantial researches in recent years supported the Arrhenius kinetic theory^2^,^7^ and indicated that recalcitrant C with higher activation energy has greater *Q*_10_ than labile C^11^,^12^,^13^,^14^,^15^,^16^. Because soil C quality generally decreases with increasing depth through mineral soil profile due to possible differences in physical and/or chemical protection from C decomposition and microbial community composition^17^, the increase in *Q*_10_ with increasing C recalcitrance, also known as the “C quality-dependent hypothesis” of *Q*_10_, has thus been tested at different depths of mineral soils^14^,^16^. In boreal and temperate forests with organic forest floor, a large amount of SOC is stored in the organic layer besides the mineral soil. The organic layer and the mineral soil have different rates of C input, accumulation, and turnover, resulting in different substrate availability and quality for C decomposition^18^,^19^. Substrate availability, besides substrate quality, could affect *Q*_10_ based on the Michaelis–Menten kinetics^2^,^20^,^21^. Consequently, the temperature sensitivity of C decomposition are likely to differ between the two soil layers^13^,^22^.

It has been suggested that *Q*_10_ of C decomposition may be latitude-dependent, with greater *Q*_10_ at higher latitude^23^,^24^. The altitudinal pattern of C decomposition with temperature change may be comparable with that caused by latitudinal gradient^14^,^22^. Therefore, elevation gradients in mountains are well recognized as “natural experiments” to explore responses of SOC to temperature changes^25^. Some studies suggested *Q*_10_ values of both labile and recalcitrant C increased along the elevation gradient in Mountain Wuyi of China^14^,^16^. On the contrary, other studies found no altitudinal trend for *Q*_10_ of C decomposition^22^,^26^. These inconsistencies may be explained by further understanding of the interactions of intrinsic (e.g. substrate availability) and extrinsic controlling factors (e.g. soil texture) of temperature sensitivity of C decomposition.

Here we collected soils from the northern slope of Mountain Changbai in Northeast China^27^ (Table 1) to investigate variations of *Q*_10_ of decomposition along the elevation gradient. Soil samples from both the organic and the mineral layers were incubated for 350 days and *Q*_10_ of decomposition was determined by the approach derived from Conant et al.^11^ (see Methods). The specific questions we aim to address in this study include: (1) Does *Q*_10_ of C decomposition of both the mineral layer and the organic layer support the “C quality-dependent hypothesis”? (2) Whether and how does *Q*_10_ of the organic layer differ from that of the mineral soil along the elevation gradient?

## Results

### Soil characteristics

**Figure 1:**
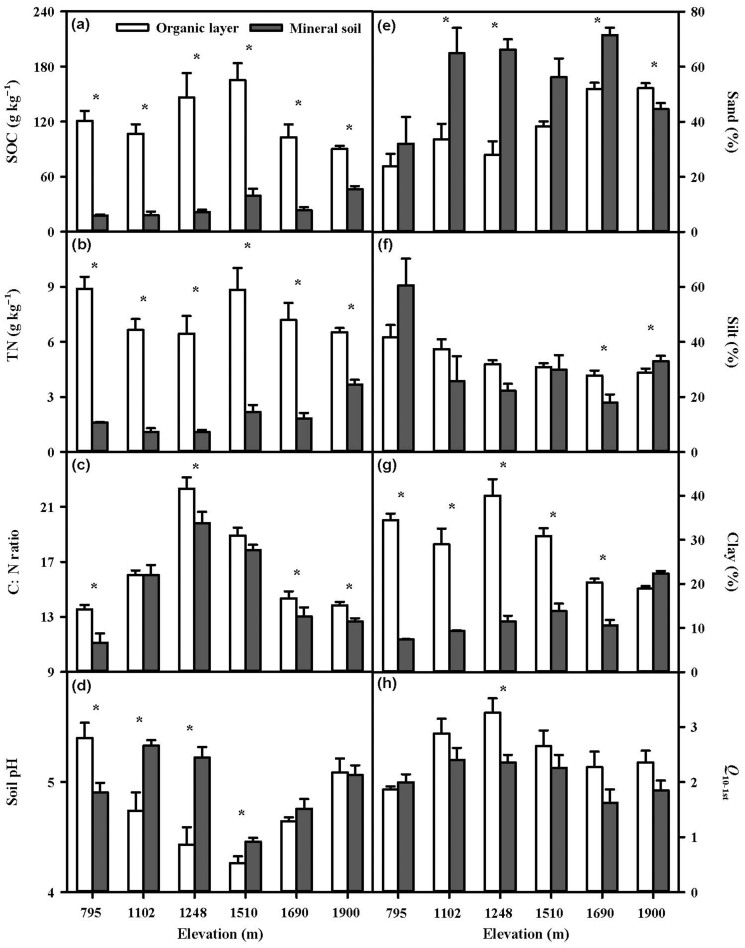
Soil properties and *Q*_10_ in the organic layer and the mineral soil along the elevation gradient (mean ± SE, n = 4). *: *p* < 0.05, which indicates significant differences between the two soil layers.

Soil organic C (SOC) and total N (TN) in the organic layer were 1.9–6.8 and 1.8–6.1 times higher (both *p* < 0.05) than those in the mineral soil across the 6 elevation sites (Fig. 1a and b, Table 2). Correspondingly, C:N ratio (*p* < 0.05) in the organic layer was higher compared to the mineral soil (Fig. 1c). In the organic layers, SOC ranged from 90.3 g kg^−1^ at 1900 m to 165.2 g kg^−1^ at 1510 m (Fig. 1a). However, TN in the organic layer was not influenced by elevation whereas its values varied from 6.5 g kg^−1^ at 1248 m to 8.9 g kg^−1^ at 795 m (Fig. 1b). SOC and TN in the mineral soil increased from 17.9 to 46.6 g kg^−1^ and from 1.1 to 3.7 g kg^−1^ along the elevation gradients, respectively. Soil pH and texture were also different among the 6 sites (all *p* < 0.05) and between the two soil layers (all *p* < 0.05 except for silt content) (Fig. 1d–g, Table 2). Soil organic layer had higher clay content and lower sand content than the mineral soil across the 6 sites (Fig. 1e and g).

### SOC decomposition

**Figure 2:**
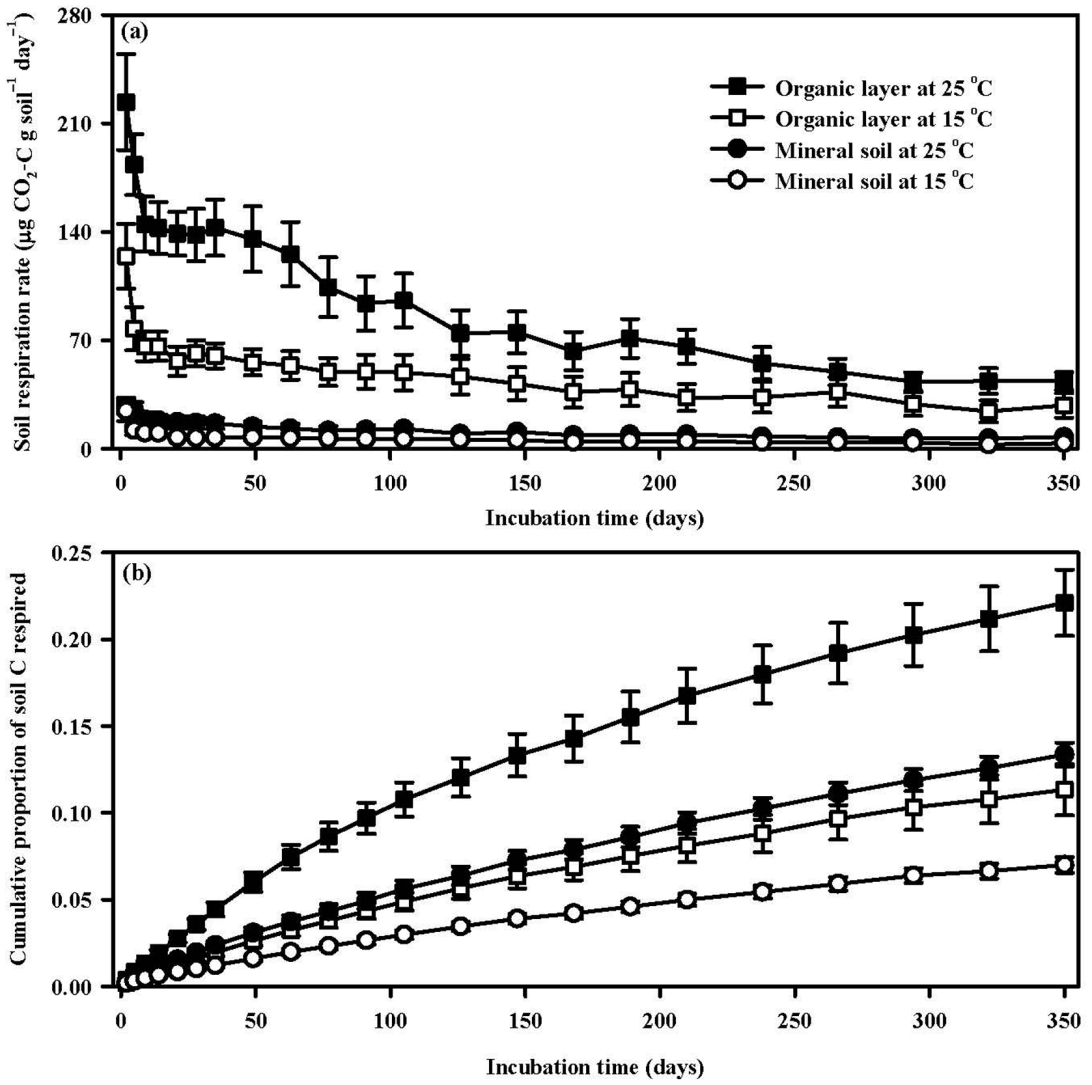
Soil respiration rate (a) and cumulative proportion of soil C respired (b) in the organic layer and the mineral soil across the 6 elevations at different incubation temperature during the incubation (mean ± SE, n = 6).

Soil respiration rates decreased by 343.8% for the organic layer and by 575.8% for the mineral layer after the 350-day incubation period at 15°C (both *p* < 0.05, Fig. 2a, Table 3). Both respiration rates and cumulative proportion of soil C respired incubated at 25°C were higher than those at 15°C during the whole incubation period (both *p* < 0.05, Fig. 2). Means of soil respiration rates over the whole incubation period was 7.6 times greater (*p* < 0.05) in the organic layer than in the mineral soil across the incubation temperatures and the elevation gradients (Fig. 2a). Consequently, the cumulative proportion of C respired after 350 days of incubation in the organic layer (22.1% at 25°C and 11.3% at 15°C, respectively) were 1.6 times higher (both *p* < 0.05) than those in the mineral soil (13.4% at 25°C and 7.0% at 15°C, respectively) (Fig. 2b). In addition, both of the two measured variables were different among sites with different elevations (both *p* < 0.05, Table 3). Soil C decomposition rates incubated at 15°C in the organic layer and the mineral soil, for example, ranged from 30.0 µg CO_2_-C g soil^−1^ day^−1^ at 1690 m to 86.9 µg CO_2_-C g soil^−1^ day^−1^ at 1510 m and from 3.2 µg CO_2_-C g soil^−1^ day^−1^ at 795 m to 12.3 µg CO_2_-C g soil^−1^ day^−1^ at 1900 m, respectively.

### Temperature sensitivity

**Figure 3:**
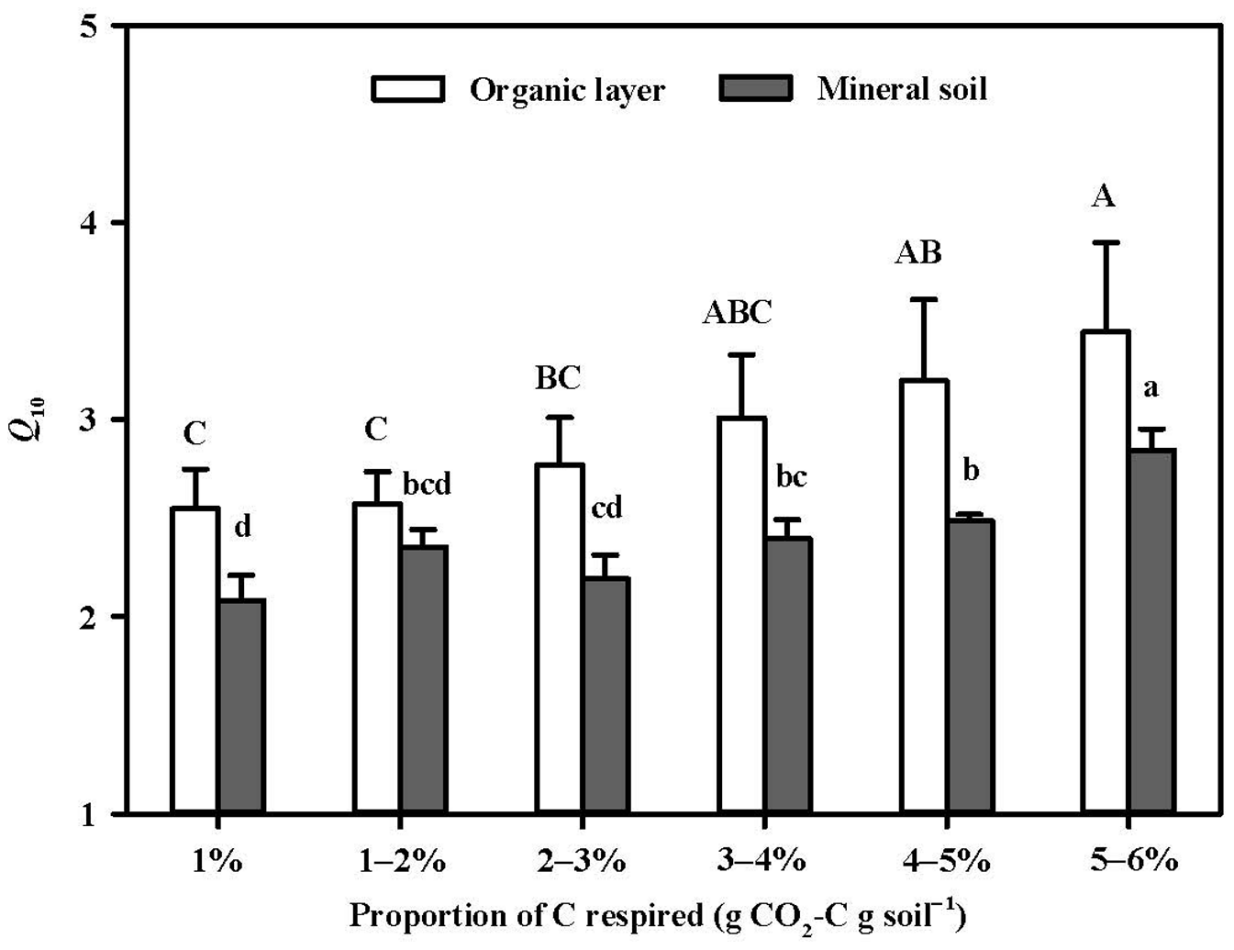
Changes in *Q*_10_ associated with proportion of soil C respired in the organic layer and the mineral soil along the elevations during the incubation (mean ± SE, n = 6). Bars with different upper- and lower-case letters are significantly different (*p* < 0.05) in the organic layer and the mineral soil, respectively.

The temperature sensitivity (*Q*_10_) of SOC decomposition increased with increasing proportion of C respired over the incubation period in both the organic layer and the mineral soil across the elevation gradient (Fig. 3). The *Q*_10_ values in the two soil layers were estimated from 1 to 6% of the proportion of respired C to initial SOC by a step of 1% and named as *Q*_10–1st_, *Q*_10–2nd_, *Q*_10–3rd_, *Q*_10–4th_, *Q*_10–5th_, or *Q*_10–6th_, respectively. *Q*_10–3rd_, for example, was determined by dividing the time taken to respire the third 1% of initial C after 2% of initial C was respired at 15°C by that at 25°C. When more than 5% and 4% of the initial SOC had been decomposed in the organic layer and the mineral soil, respectively, *Q*_10_ of the 1% SOC (*Q*_10–5th_ for the organic layer and *Q*_10–4th_ for the mineral layer) began to be different from *Q*_10–1st_ significantly (Fig. 3). *Q*_10_ values of the organic layer (2.5–3.4) were always higher (all *p* < 0.05 except for *Q*_10–2nd_) compared to those of the mineral soil (2.1–2.8) during the incubation (Fig. 3, Table 2). There was no altitudinal trend of *Q*_10_ (*Q*_10-1st_–*Q*_10-6th_) in either soil layer. For example, the highest *Q*_10–1st_ value of the organic layer and the mineral soil occurred at 1248 m (3.3) and 1102 m (2.4), respectively (Fig. 1h). In addition, elevation also interacted with soil layer to influence *Q*_10_ (Table 2).

## Discussion

The alteration of soil C quality can arise through the progressive depletion of labile C and the increasing contribution of recalcitrant C to SOC decomposition during the long-term incubation^11^,^14^ compared to the relatively short incubation duration^4^. In this 350-day incubation study, the curves of changes in SOC decomposition rates (Fig. 2a) revealed that respiration declined sharply in the first 3 weeks and then started to settle and reduce slowly in both the organic layer and the mineral soil. This observed pattern was consistent with previous long-term laboratory incubation studies^11^,^14^,^15^,^16^. The enhancement of *Q*_10_ over incubation time in both the organic layer (from 2.5 in *Q*_10–1st_ to 3.4 in *Q*_10–6th_) and the mineral soil (from 2.1 in *Q*_10–1st_ to 2.8 in *Q*_10–6th_) (Fig. 3) suggested that the temperature sensitivity of more labile C was lower than that of more recalcitrant C. This result could be well explained by the Arrhenius equation (*k* = *A* exp(–*E_a_*/*RT*)), where *k* is the reaction rate of C decomposition, *A* is the frequency or pre-exponential factor (the theoretical reaction rate at *E_a_* = 0), *E_a_* is the activation energy of C components (J mol^−1^), *R* is the gas constant (8.314 J mol^−1^ K^−1^), and *T* is the absolute temperature (in Kelvin). It indicated that higher *E_a_* was required for decomposition of more recalcitrant C due to less reaction of stabilized substrates^2^,^7^. *Q*_10_, defined as the factor by which *k* increases with increasing temperature by 10°C, should increase with increasing *E_a_* and C recalcitrance. Therefore, our results support the “C quality-dependent hypothesis”, which has been proven in temperate grassland^11^,^15^, subtropical forest^14^,^16^, boreal forest^13^, and cropland^12^. Our findings in the temperate forest, together with these previous reports point to the critical role of the relatively recalcitrant C fraction with higher amounts and higher temperature sensitivity in affecting C balance and cycling under further climate warming.

We found constantly higher *Q*_10_ values in the surface organic layer than the subsurface mineral soil during the incubation (Fig. 3), which was distinct from previous findings of increasing *Q*_10_ with soil depths in the mineral soil profiles^14^,^16^,^17^. In addition to Arrhenius kinetics, *Q*_10_ of decomposition may be affected by Michaelis–Menten kinetics simultaneously. We believe this finding may be explained by the Michaelis–Menten kinetics (*k* = *V*_max_ × [*S*]/(*K*_m_ + [*S*]), where *k* is the reaction rate, *V*_max_ is the maximum rate of enzymatic activity at a given temperature, [*S*] is the substrate availability (substrate concentration at active site of enzyme), and *K*_m_ is the Michaelis–Menten constant, representing the affinity of enzymes for the substrates expressed as the substrate concentration at which the reaction rate equals *V*_max_/2. Both *V*_max_ and *K*_m_ are temperature dependent and their temperature sensitivities can neutralize each other, called “cancelling effect”. When the substrate is least limiting ([*S*] ≫ *K*_m_), this “cancelling effect” is eliminated, causing higher *Q*_10_. Therefore, the higher *Q*_10_ values in the organic layer than in the mineral soil could be due to the reduction in the “cancelling effect” between *V*_max_ and *K*_m_ by the significantly higher C availability in the organic layer^13^,^20^.

The *Q*_10_ values observed in both the organic layer and the mineral soil during the incubation (Fig. 3) were larger than 2.0, which has been typically used as *Q*_10_ value of different SOC pools in current soil C models^3^. Therefore, care should be taken when applying this generally used Q_10_ value to different ecosystems. More importantly, various *Q*_10_ values in different soil C pools should be considered to be incorporated into soil C models for better simulation of C decomposition in these forests. In particular, soil organic layer and mineral layer should have different Q_10_ values when modeling temperate forest with thick organic layers. In addition, *Q*_10_ values only reflect the relative changes in decomposition rates as a function of temperature. The absolute amounts of C that can be released from soils are the most important values to be predicted under climate change. Therefore, both the absolute and relative rates in response to temperature change should be considered in combination^2^,^28^. In this study, the high amount of SOC and *Q*_10_ of C decomposition in the organic layer (Fig. 1a and 3) could have additive effects on C release, suggesting the extreme importance of the organic layer.

We did not find a clear trend of *Q*_10_ along the elevation gradient, which is in accordance with findings in temperate and boreal forests^22^,^26^, whereas increase of *Q*_10_ with increasing elevation was observed in subtropical forests^14^,^16^. These contradictory results could be explained by the different controls of *Q*_10_ under different conditions. Soil properties have been postulated to play roles in regulating *Q*_10_ due to their influences on decomposability of soil C^15^. For example, in mineral soil of subtropical forests, Xu et al.^14^ and Wang et al.^16^ found that both microbial biomass C and soil C:N ratio increased with increasing elevation, which could result in the positive relationship of *Q*_10_ with elevation. While in our study, SOC, TN, and soil C:N ratio did not show any altitudinal patterns in either the organic layer or the mineral soil (Fig. 1a and b), which may be the reason why no clear trend of *Q*_10_ was observed along the elevation gradient. For the organic soils in Niklińska and Klimek^26^, we re-calculated soil C:N ratios based on the soil C and N concentrations in their paper and found no effect of elevation on C:N ratio. Therefore, how elevation may affect of *Q*_10_ decomposition actually depends on how elevation influences quality and quantity of soil organic matter and/or activity and abundance of decomposers.

In conclusion, our results suggested the organic layer had higher *Q*_10_ than the mineral soil, which is consistent with the Michaelis-Menten kinetics. This highlights the necessity of separating the organic layer from the mineral soil to explore the responses of soil C decomposition to temperature. When the two layers were considered separately, the Arrhenius kinetic theory was supported by our results which found decomposition *Q*_10_ increased with decreasing soil C lability. Because the SOC pool of the organic layer in temperate forests is large, its higher temperature sensitivity highlights its importance in C cycling under global warming. The different *Q*_10_ values observed in the two soil layers should be incorporated into soil C models for better prediction of responses of C decomposition to temperature change in temperate ecosystems.

## Methods

### Site description and soil sampling

The study was conducted on the northern slope of Mountain Changbai in Northeast China (41°42′–42°25′N, 127°42′–128°17′E). Mean annual temperature declined from 2.0 to −3.7°C and mean annual precipitation increased from 702 to 1038 mm with increasing altitude from 795 to 1900 m. Forest types were especially distinct due to climatic changes along the elevation gradient. A 20 × 20 m^2^ plot was set at each of the six major vegetation types with different elevation. The detailed site descriptions were shown in Table 1.

Soil samples of the organic layer and the upper 10 cm of the mineral soil were collected at 4 points randomly within each plot in September 2011. The organic layer was distinguished from the mineral soil by its morphology (including soil color, texture, and consistency). Soils were black or dark brown in color and less friable in the organic layer compared to the mineral soil. In addition, the depths of the organic layer were 3.3–4.9 cm across the 6 elevations (Table 1). Each fresh soil sample was sieved with a 2-mm sieve to remove rocks and root materials, thoroughly hand-mixed, and divided into two subsamples. One subsample was stored at 4°C until incubation began. The other subsample was air-dried to measure soil properties. SOC, total nitrogen (TN), and C:N ratio were determined on oven-dried (60°C) soil subsample by an elemental analyzer (VARIO EL III, Germany). Soil pH was measured with a Calomel electrode at 1:5 soil-to-water ratio. Soil texture was measured by the pipette method^29^. Soil water content in both soil layers and field water capacity of mineral soil samples were also determined.

### Soil incubation

Fresh soil samples of the organic layer (50 g with original soil moisture) and the mineral layer (150 g with 60% of field capacity) were incubated in 1 L Mason jars at 15 and 25°C, respectively. Empty jars without soil samples were used as controls. The soil samples were pre-incubated for five days before measurements of SOC decomposition by the alkali absorption method^14^. In detail, soil respired CO_2_ was trapped by 30 ml NaOH (1M) solution in an open vial placed in each sealed jar. Samples were taken 22 times (2, 5, 9, 14, 21, 28, 35, 49, 63, 77, 91, 105, 126, 147, 168, 189, 210, 238, 266, 294, 322, 350 days after the incubation) with different intervals. The amount of respired CO_2_ was determined by titration of NaOH with 1 M HCl to pH = 8.3 in the presence of BaCl_2_. Samples were flushed with compressed air to allow replenishment of O_2_ and were remoistened to maintain moisture after each interval. The cumulative proportion of soil C respired at a given sampling time was calculated as the summed amounts of C respired before the given sampling time divided by SOC.

### Temperature sensitivity

Temperature sensitivity (*Q*_10_) of SOC decomposition during the incubation was calculated using the method developed by Conant et al.^11^: 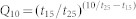
where *t*_15_ and *t*_25_ are the time required to respire a given amount of soil C during the incubation at 15 and 25°C, respectively.

### Statistical analysis

Shapiro-Wilk and Levene's tests were used to analyze the data for normality and homogeneity of variance, and if necessary, data were transformed by the square root or logarithmic transformation. Two-way ANOVAs were used to analyze the effects of soil layer and elevation on the *Q*_10_ values with a given proportion of cumulative C respired during the incubation and soil characteristics (SOC, TN, C:N ratio, pH value, and texture). The differences between the organic and the mineral layers at each site were also determined by paired *t* tests. The cumulative proportion of C respired and soil respiration rate during the incubation were analyzed by repeated-measures ANOVA and tested for sampling time, soil layer, elevation, and their interactions. All statistical analyses were performed by SAS 9.2 software (SAS Institute Inc., Cary, NC, USA).

## Author Contributions

E.B. and W.X. designed the experiment. W.X. conducted the measurements, data analyses and wrote the manuscript. W.L., P.J. and H.W. assisted with the experiments. E.B. and W.X. reviewed the manuscript.

## Figures and Tables

**Table 1:** Sites description

Elevation (m)	Position	MAT (°C)	MAP (mm)	Tree category	Vegetation type	Dominant species	Soil type	The depth of organic layer§
795	42°23′N, 128°05′E	2.0	702	Mixed coniferous broad-leaved forest	Deciduous broadleaf and evengreen needleleaf	*Pinus koraiensis Tilia amuresis Fraxinus mandshurica*	Dark brown forest soil	4.9 ± 0.2a
1102	42°11′N, 128°09′E	0.4	783	Broad-leaved Korean pine forest	Deciduous broadleaf and evengreen needleleaf	*Pinus koraiensis Picea jezoensis*	Brown coniferous forest soil	3.7 ± 0.2b
1248	42°08′N, 128°07′E	−0.3	824	Korean pine-spruce-fir forest	Evengreen needleleaf	*Abies nephrolepis Picea jezoensis Larix gmellini*	Brown coniferous forest soil	3.6 ± 0.3b
1510	42°05′N, 128°04′E	−1.7	904	Dark coniferous spruce-fir forest	Evengreen needleleaf	*Picea jezoensis Abies nephrolepis*	Brown coniferous forest soil	3.3 ± 0.1b
1690	42°04′N, 128°03′E	−2.6	964	Ermans birch-spruce-fir forest	Evengreen needleleaf	*Picea jezoensis Abies nephrolepis Betula ermanii*	Brown coniferous forest soil	3.6 ± 0.6b
1900	42°03′N, 128°04′E	−3.7	1038	Ermans birch forest	Deciduous broadleaf	*Betula ermanii*	Soddy forest soil	3.9 ± 0.4b

^§^Data are mean ± SE (n = 4). Lowercase letters next to data indicate significant differences (*p* < 0.05) in the depths of the organic layer among the 6 elevations determined by *t* test.

**Table 2:** Results (*F*-values) of two-way ANOVA for soil properties and *Q*_10_. *: *p* < 0.05, ns: *p* > 0.05

					Soil texture								
Source of variance	SOC	TN	C:N ratio	pH	Sand	Silt	Clay	*Q*_10–1st_	*Q*_10–2nd_	*Q*_10–3rd_	*Q*_10–4th_	*Q*_10–5th_	*Q*_10–6th_
Soil layer (SL)	201.5*	247.4*	18.6*	10.6*	34.5*	0.3ns	244.2*	13.5*	2.6ns	15.8*	20.3*	17.2*	8.2*
Elevation (E)	3.6*	2.9*	71.7*	16.4*	8.3*	8.6*	7.1*	5.8*	1.3ns	2.4ns	3.1*	5.1*	4.8*
SL × E	3.6*	3.1*	1.4ns	9.8*	4.8*	3.1*	21.4*	1.2ns	2.6*	4.4*	9.1*	6.5*	5.1*

**Table 3:** Results (*F*-values) of repeated-measures ANOVA for soil respiration rate and cumulative proportion of soil C respired among the 22 sampling times during the incubation.*: *p* < 0.05, ns: *p* > 0.05

Source of variance	Sampling time	Incubation temperature (T)	Soil layer (SL)	Elevation (E)	T × SL	T × E	SL × E	T × SL × E
Soil respiration rate	271.4*	159.2*	848.4*	28.7*	100.1*	2.5*	20.4*	1.7ns
Cumulative proportion of soil C respired	1899.9*	219.8*	141.8*	11.9*	23.9*	1.5ns	4.2**	0.6ns

## References

[b1] Schmidt M. W. I. *et al.* Persistence of soil organic matter as an ecosystem property. Nature 478, 49–56 (2011). 21979045 10.1038/nature10386

[b2] Davidson E. A. & Janssens I. A. Temperature sensitivity of soil carbon decomposition and feedbacks to climate change. Nature 440, 165–173 (2006). 16525463 10.1038/nature04514

[b3] Melillo J. M. *et al.* Soil warming and carbon-cycle feedbacks to the climate system. Science 298, 2173–2176 (2002). 12481133 10.1126/science.1074153

[b4] Fang C., Smith P., Moncrieff J. B. & Smith J. U. Similar response of labile and resistant soil organic matter pools to changes in temperature. Nature 433, 57–59 (2005). 15635408 10.1038/nature03138

[b5] Conant R. T. *et al.* Temperature and soil organic matter decomposition rates–synthesis of current knowledge and a way forward. Glob. Change Biol. 17, 3392–3404 (2011).

[b6] Hamdi S., Moyano F., Sall S., Bernoux M. & Chevallier T. Synthesis analysis of the temperature sensitivity of soil respiration from laboratory studies in relation to incubation methods and soil conditions. Soil Biol. Biochem. 58, 115–126 (2013).

[b7] Bosatta E. & Ågren G. I. Soil organic matter quality interpreted thermodynamically. Soil Biol. Biochem. 31, 1889–1891 (1999).

[b8] Cox P. M., Betts R. A., Jones C. D., Spall S. A. & Totterdell I. J. Acceleration of global warming due to carbon-cycle feedbacks in a coupled climate model. Nature 408, 184–187 (2000). 11089968 10.1038/35041539

[b9] Conen F., Leifeld J., Seth B. & Alewell C. Warming mineralises young and old soil carbon equally. Biogeosciences 3, 515–519 (2006).

[b10] Liski J., Ilvesniemi H., Mäkelä A. & Westman C. J. Temperature dependence of old soil organic matter. Ambio 29, 56–57 (2000).

[b11] Conant R. T. *et al.* Sensitivity of organic matter decomposition to warming varies with its quality. Glob. Change Biol. 14, 868–877 (2008).

[b12] Hartley I. P. & Ineson P. Substrate quality and the temperature sensitivity of soil organic matter decomposition. Soil Biol. Biochem. 40, 1567–1574 (2008).

[b13] Karhu K. *et al.* Temperature sensitivity of organic matter decomposition in two boreal forest soil profiles. Soil Biol. Biochem. 42, 72–82 (2010).

[b14] Xu X., Zhou Y., Ruan H., Luo Y. & Wang J. Temperature sensitivity increases with soil organic carbon recalcitrance along an elevational gradient in the Wuyi Mountains, China. Soil Biol. Biochem. 42, 1811–1815 (2010).

[b15] Haddix M. L. *et al.* The role of soil characteristics on temperature sensitivity of soil organic matter. Soil Sci. Soc. Am. J. 75, 56–68 (2011).

[b16] Wang G., Zhou Y., Xu X., Ruan H. & Wang J. Temperature sensitivity of soil organic carbon mineralization along an elevation gradient in the Wuyi Mountains, China. PLoS ONE 8, e53914, 10.1371/journal.pone.0053914 (2013). 23342038 10.1371/journal.pone.0053914 PMC3544745

[b17] Fierer N., Allen A. S., Schimel J. P. & Holden P. A. Controls on microbial CO2 production: a comparison of surface and subsurface soil horizons. Glob. Change Biol. 9, 1322–1332 (2003).

[b18] Trumbore S. E. & Harden J. W. Accumulation and turnover of carbon in organic and mineral soils of the BOREAS northern study area. J. Geophys. Res. Atmospheres 102, 28817–28830 (1997).

[b19] Côté L., Brown S., Paré D., Fyles J. & Bauhus J. Dynamics of carbon and nitrogen mineralization in relation to stand type, stand age and soil texture in the boreal mixedwood. Soil Biol. Biochem. 32, 1079–1090 (2000).

[b20] Gershenson A., Bader N. E. & Cheng W. Effects of substrate availability on the temperature sensitivity of soil organic matter decomposition. Glob. Change Biol. 15, 176–183 (2009).

[b21] Fissore C., Giardina C. P. & Kolka R. K. Reduced substrate supply limits the temperature response of soil organic carbon decomposition. Soil Biol. Biochem. 67, 306–311 (2013).

[b22] Schindlbacher A. *et al.* Temperature sensitivity of forest soil organic matter decomposition along two elevation gradients. J. Geophys. Res. 115, G03018, 10.1029/2009JG001191 (2010).

[b23] Fierer N., Colman B. P., Schimel J. P. & Jackson R. B. Predicting the temperature dependence of microbial respiration in soil: A continental-scale analysis. Glob. Biogeochem. Cycles 20, GB3026, 10.1029/2005GB002644 (2006).

[b24] Vanhala P. *et al.* Temperature sensitivity of soil organic matter decomposition in southern and northern areas of the boreal forest zone. Soil Biol. Biochem. 40, 1758–1764 (2008).

[b25] Körner C. The use of ‘altitude' in ecological research. Trends Ecol. Evol. 22, 569–574 (2007). 17988759 10.1016/j.tree.2007.09.006

[b26] Niklińska M. & Klimek B. Effect of temperature on the respiration rate of forest soil organic layer along an elevation gradient in the Polish Carpathians. Biol. Fertil. Soils 43, 511–518 (2007).

[b27] Tian Q., He H., Cheng W. & Zhang X. Pulse-dynamic and monotonic decline patterns of soil respiration in long term laboratory microcosms. Soil Biol. Biochem. 68, 329–336 (2014).

[b28] Sierra C. A. Temperature sensitivity of organic matter decomposition in the Arrhenius equation: some theoretical considerations. Biogeochemistry 108, 1–15 (2012).

[b29] Gee G. M. & Bauder J. W. Methods of Soil Analysis, Part I: Physical and Mineralogical Methods. (American Society of Agronomy, Inc. and Soil Science Society of America, Inc.,USA ), pp. 383–411 (1986).

